# Association between psychological distress and quality of life among caregivers of children with cerebral palsy: A cross-sectional study

**DOI:** 10.6026/973206300212625

**Published:** 2025-08-31

**Authors:** Priti Nisheet Agni, Prasad Muley, Geeta Bhatt

**Affiliations:** 1 Department of Physiotherapy, Parul Institute of Doctoral Studies, Parul University, Vadodara, Gujarat, India; 2 Department of Pediatrics, Parul Institute of Doctoral Studies, Parul University, Vadodara, Gujarat, India; 3 Department of Neuro Physiotherapy, KJ Somaiya College of Physiotherapy, Mumbai, Maharashtra, India

**Keywords:** Depression, stress, anxiety, caregivers, quality of life

## Abstract

Caring for a Cerebral Palsy (CP) child is associated with psychological and physical demands leading to elevated levels of stress, anxiety, depression. This cross-sectional study aims to find the association between stress, anxiety, depression and quality of life among caregivers of children with Cerebral Palsy (CP) and was conducted among 194 caregivers of children with CP. DASS-21, WHOQOL BREF scales were used to evaluate stress, anxiety and depression and Quality of life (QOL), respectively, while the functional capabilities were assessed through the Gross Motor Function Classification System (GMFCS). Data shows a significant negative correlation between DASS and the WHOBREF Scale. Higher levels of stress, anxiety and depression were significantly associated with lower scores across all domains of QOL (p < 0.05). The study highlights a strong association of stress, anxiety, depression amongst caregivers and QOL.

## Background:

Cerebral palsy (CP) is a group of permanent disorders of the development of movement and posture, attributed to non-progressive disturbances that occurred in the developing fetal or infant brain [1]. With an estimated prevalence of two to three per 1,000 live births, cerebral palsy (CP) continues to be the most prevalent motor disability in children worldwide [2]. Cerebral palsy is frequently accompanied by abnormalities in sensation, perception, cognition, behavior and communication1. It usually manifests with comorbidities like intellectual disabilities, musculoskeletal issues and epilepsy, requiring multifaceted, long-term care [3]. These challenges necessitate lifelong caregiving and comprehensive management, primarily provided by family members, most often mothers, who assume the role of primary caregivers. Providing care for someone with cerebral palsy (CP) entails a lot of unpaid time, effort and resources over a long period. Caregivers of children with cerebral palsy often experience poor sleep quality and psychological distress, significantly impacting their overall well-being [4]. To emphasize the level of involvement of caregivers in the care of patients with CP and their stress, they have often been referred to as second victims of the disease. We need to acknowledge that they have to take on this role under sudden and extreme circumstances, with minimal preparation and little guidance and support from healthcare systems [5]. Numerous physically, emotionally, socially and financially taxing tasks are performed by caregivers [6]. Because of this ongoing strain, caregivers are frequently more susceptible to psychological problems like anxiety, depression and chronic stress [7]. Unchecked caregiving stress can negatively affect a caregiver's health and relationships with others, which can ultimately make it more difficult for them to give the child the critical support they need [8]. In addition to lowering one's well-being, the emotional toll of providing care can also lead to a vicious cycle that makes caring for children even more difficult and lowers the standard of care [9]. According to research, parents of children with cerebral palsy (CP) have greater rates of anxiety and depression than parents of children without disabilities [10, 11]. Caregivers may have to reduce working hours or quit employment altogether, thereby increasing financial strain. A recent study in Saudi Arabia highlighted multiple demographic and psychosocial factors influencing caregiver quality of life among families of children with cerebral palsy [12]. Furthermore, they often receive inadequate social recognition and emotional support, which increases their vulnerability to psychological distress. Factors affecting quality of life in mothers of children with cerebral palsy vary across cultural contexts, with Iranian studies emphasizing the role of social support and coping strategies [13]. Psychological distress among caregivers, which includes stress, anxiety and depression, is a well-documented phenomenon. Studies have reported that up to 60% of caregivers of children with CP experience moderate to severe psychological symptoms [7]. These emotional difficulties are not merely transient but may persist and intensify over time, affecting both the caregiver's physical and mental health. One of the critical aspects impacted by psychological distress is the quality of life (QoL) of the caregiver. Caregivers of children with CP often report lower QoL across multiple domains, physical health, psychological well-being, social relationships and environmental satisfaction, compared to parents of typically developing children [6, 8]. Understanding the interrelationship among caregiver stress, mental health outcomes and quality of life is crucial for developing effective, family-centred support systems [6, 8]. While the burden of caregiving has been acknowledged, there is still a significant gap in understanding the full extent to which stress, anxiety and depression interrelates and impact the overall quality of life among this population, especially in diverse cultural and healthcare settings [8, 10 and 11]. Therefore, it is of interest to explore the association between psychological distress (stress, anxiety and depression) and quality of life in caregivers of children with cerebral palsy.

## Methodology:

This cross-sectional study involved 194 caregivers of children suffering from Cerebral Palsy in Mumbai and received ethical clearance from K J Somaiya Medical College and Hospital (ECR/138/Inst/MH/2013/RR-19). Participants were recruited from families accessing specialized services in rehabilitation facilities from February 2023 to January 2025. Sample size calculation was based on a 7.9% prevalence rate of mental health strain among caregivers, with a 95% confidence limit and a 5% significance level, resulting in a calculated sample size of 194 [14]. Subjects were recruited through convenience sampling and screened according to the inclusion and exclusion criteria. The study population consisted of informal, unpaid caregivers of all genders, aged between 20 and 50 years. Inclusion criteria required caregivers to be cognitively intact (with a Mini-Mental State Examination (MMSE) score greater than 23) and actively involved in providing primary care to the child. For the children with Cerebral Palsy, inclusion criteria stipulated that they must have a diagnosis according to the ICD-10 and be aged between 2 and 18 years. Caregivers with chronic medical conditions or those unwilling to participate were excluded from the study. All eligible participants provided informed consent before being enrolled. Psychological distress was measured using the DASS-21 scale for stress, anxiety and depression [15, 16]. The WHOQOL-BREF questionnaire evaluated quality of life across physical health, psychological well-being, social relationships and environmental factors [17]. Motor impairment severity was classified using GMFCS [18]. The caregivers were informed about the study's main purpose, the importance of their contribution, the confidentiality of their responses, the estimated time required for completing the survey and the issues addressed within the questionnaire. Following this information, caregivers voluntarily participated in the survey. Detailed interviews were conducted, using a specially designed questionnaire to gather socio-demographic characteristics of both caregivers and their children. Information collected included the child's age and gender, the caregiver's age, gender, education, annual family income, hours spent on caregiving, the number of other dependent family members and any additional help received in caregiving. The type of CP and the child's motor function level were recorded. Out of 205 individuals approached, 4 did not meet the inclusion criteria and 4 declined to participate. Three questionnaires were rejected due to incomplete data. Ultimately, 194 caregivers participated without compensation.

## Results:

Categorical variables were expressed as numbers and percentages, while continuous variables were presented as mean ± standard deviation (SD). Spearman's rank correlation coefficient was used for correlation analysis between variables. To assess associations between categorical dependent and independent variables, the Chi-square test or Fisher's exact test was employed. The final analysis was performed using the Statistical Package for the Social Sciences (SPSS) software version 21.0. A p-value of less than 0.05 was considered statistically significant. This study included 194 caregivers of children with diagnosed Cerebral Palsy. Amongst them were 139 (71.64%) females and 55 (28.35%) males. The mean age of caregivers in years was 38.55 ± 7.281. The mean age of Cerebral Palsy patients in years was 10.45 ± 2.996. The caregivers dedicated approximately 11 hours per day to caregiving responsibilities (Table 1 - see PDF). This extensive commitment suggests a significant emotional and physical toll, which was further examined through the Depression, Anxiety and Stress Scale (DASS). The results revealed high levels of Stress (18.93 ± 9.534), Anxiety (12.88 ± 8.666), Depression (18.29 ± 10.157) and caregiver burden (22.15 ± 6.44) (Figure 1 - see PDF). Caregivers' quality of life was substantially compromised across all domains: Physical health, 44.02 ± 23.072; Psychosocial health, 44.11 ± 24.774; Social relationships, 36.25 ± 26.152; Environment, 46.88 ± 25.277 (Figure 2 - see PDF). Table 2-4 (see PDF) further refines this analysis by exploring the correlation between depression, anxiety and stress with QOL. A negative correlation is observed between depression, anxiety and stress with physical health, psychological, social and environmental domains of QOL. This suggests higher levels of depression, anxiety and stress were associated with lower scores across all domains of QOL (p < 0.05). A positive correlation is seen between GMFCS and DASS, suggesting that higher GMFCS levels, caregivers are likely to experience increased psychological distress (DASS: anxiety, stress and depression) (Table 5 - see PDF). A negative correlation is seen between GMFCS and all four domains of the WHO-BREF scale, suggesting that higher GMFCS levels, caregivers will have a poor quality of life (Table 6 - see PDF).

## Discussion:

This cross-sectional study explored the complex interrelationship between psychological distress (stress, anxiety and depression) and quality of life (QoL) among caregivers of children with cerebral palsy (CP), while also examining how the severity of the child's motor impairment (GMFCS level) contributes to these outcomes. The findings affirm that caregiving for a child with CP significantly compromises caregiver well-being. A detailed correlation analysis (Tables 2-4 - see PDF) indicates that higher levels of depression, anxiety and stress, as measured by the DASS-21, are significantly associated with lower QoL across all domains of the WHOQOL-BREF (p < 0.001). Depression was inversely associated with physical health (r = -0.649), psychosocial well-being (r = -0.664), social relationships (r = -0.612) and the environmental domain (r = -0.690), all with p-values < 0.001, highlighting the pervasive impact of depressive symptoms on caregivers' well-being. Depression adversely affects physical health by reducing motivation, energy, sleep quality and physical activity, which are crucial for managing the demanding physical tasks involved in caregiving [19]. This association is particularly concerning as caregiving often entails physically strenuous tasks such as lifting, bathing and assisting with mobility. Our results support previous findings by Raina *et al.* and Ryan *et al.* who reported that depressive symptoms in caregivers are linked to chronic fatigue, somatic complaints and reduced health-seeking behavior [20, 21]. Maternal depression and anxiety have a significant association with the quality of life of children with cerebral palsy, highlighting the bidirectional relationship [22]. The psychosocial domain demonstrated correlation with depression (r = -0.664), indicating that depressive symptoms erode caregivers' inner resilience, emotional coping and overall psychological balance [23]. The social relationships domain was also significantly affected (r = -0.612), suggesting that depression contributes to social withdrawal and reduced interaction [24]. The strongest association was found between depression and the environmental domain (r = -0.690), aligning with findings by Vadivelan *et al.* and King *et al.* [25, 26]. Anxiety was significantly negatively correlated with physical health (r = -0.672), psychosocial health (r = -0.695), social relationships (r = -0.654) and environmental context (r = -0.718). These findings align with previous studies that demonstrate how anxiety disorders in caregivers are associated with somatic complaints, cardiovascular strain, poor sleep and chronic fatigue [23, 27]. Physically, the toll of caregiving, coupled with persistent worry about the child's future and uncertainty about care availability, may lead to an ongoing state of hypervigilance, exacerbating the physiological symptoms of anxiety. The negative correlation with psychosocial well-being (r = -0.695) implies that anxiety impairs emotional functioning and existential satisfaction, which are the core components of WHOQOL-BREF's psychological domain. These results echo those of those who found high anxiety levels among caregivers of children with CP, particularly among mothers, with significant impairment in psychological and social domains [11, 24]. Anxiety also contributed to deterioration in social relationships (r = -0.654). Caregivers experiencing anxiety may avoid social gatherings, limit communication, or experience interpersonal tension, contributing to emotional isolation and loss of social support networks. The environmental domain showed the strongest association (r = -0.718), suggesting that caregivers with heightened anxiety perceive greater barriers in their environment such as inadequate transportation, inaccessible healthcare, financial stress, or lack of respite services factors previously identified by King *et al.* (2012) as major stressors in families with children who have CP [26]. Similar to anxiety, stress was strongly negatively associated with all four WHOQOL-BREF domains: physical health (r = -0.701), psychosocial health (r = -0.725), social relationships (r = -0.682) and environment (r = -0.733). These findings reflect the all-encompassing impact of chronic stress, which undermines not only physical and psychological health but also relational functioning and satisfaction with life conditions. The strongest correlation between stress and the environmental domain suggests that environmental limitations are perceived more acutely when caregivers are under prolonged stress. Financial concerns, perceived inadequacies in healthcare access and lack of assistive services exacerbate stress and create a feedback loop of burnout and helplessness, as shown in prior research [20, 25]. The psychosocial impact of stress (r = -0.725) is well supported by literature describing how prolonged exposure to caregiving stress can lead to emotional dysregulation, irritability, low self-esteem and even suicidal ideation in extreme cases [6, 28]. This is particularly relevant in caregivers of children with CP, where caregiving is often a lifelong responsibility, with minimal personal respite or professional support. Stress also significantly impacted physical health (r = -0.701), indicating that stress manifests somatically through chronic fatigue, hypertension and disrupted sleep symptoms frequently reported by caregivers in high-burden environments [19]. The social relationship domain (r = -0.682) was similarly affected, likely due to emotional exhaustion, relationship conflict and limited time for personal or social engagement. GMFCS level was positively correlated with depression (r = 0.443), anxiety (r = 0.375) and stress (r = 0.408), all with p < 0.001, indicating that more severe motor impairments are associated with worse psychological outcomes in caregivers. As GMFCS levels increase, children typically require extensive assistance with basic activities of daily living, frequent medical consultations and specialized equipment or therapy factors that intensify caregiver responsibilities [3]. The cumulative physical, emotional and financial toll associated with caring for a child with GMFCS level IV or V is often overwhelming and may lead to chronic fatigue, emotional burnout and reduced coping capacity [22, 29]. The significant association between GMFCS and depression (r = 0.443) in particular underscores the emotional toll of long-term caregiving for children with profound physical limitations. Caregivers may feel helpless, uncertain about the child's future, or socially isolated due to the demanding nature of the care, leading to a greater risk for mood disturbances. Moreover, the observed correlations with anxiety and stress emphasize the anticipatory worry, constant vigilance and logistical challenges inherent in managing severe CP cases. The present study demonstrated a significant negative correlation between the GMFCS level and all domains of caregiver quality of life. Higher GMFCS levels were associated with poorer physical health (r = -0.284), psychosocial well-being (r = -0.274), social relationships (r = -0.289) and environmental satisfaction (r = -0.305). These findings are consistent with previous research emphasizing the link between greater child disability and reduced QoL [23, 27]. Greater motor disability often requires more intensive, around-the-clock care-including feeding, toileting, mobility support and frequent medical appointments-placing substantial physical and emotional demands on caregivers [11]. Psychologically, caregivers of children at higher GMFCS levels report greater stress and helplessness, particularly due to fears about the child's future and their long-term caregiving capacity [24]. Social participation is also hindered, as severe functional limitations often restrict family outings, socialization and time for self-care, leading to social isolation. Additionally, higher GMFCS levels correlate with greater reliance on assistive devices, transport barriers and home modifications, contributing to lower satisfaction within the environmental domain [26]. These results emphasize the need for tailored caregiver support strategies, particularly for families of children with GMFCS levels IV and V, including access to home-based therapies, respite care, assistive services and caregiver-focused psychological interventions. Taken together, the results paint a poignant picture of the difficulties caregivers face in managing both their responsibilities and their well-being. These results are consistent with past studies that found caregivers of children with severe cerebral palsy are more likely to experience mental health issues and poor health outcomes [6, 8]. Recent research confirms that caregiving leads to notable psychosocial burden and diminished quality of life in primary caregivers of children with cerebral palsy [30]. The negative effects on the caregiver's health and quality of life may be made worse by the combined strain of juggling caregiving duties and psychological stressors. The multidimensional burden borne by caregivers in this study underscores the necessity of multidisciplinary, family-centered approaches. Health professionals should be trained to not only address the child's rehabilitation needs but also to assess and manage caregiver distress. Tailored educational sessions, peer support groups and better access to therapy services can help empower caregivers and mitigate their stress. Prioritizing caregiver well-being is not only essential for improving their quality of life but also beneficial for the individuals they care for. Health systems should recognize the caregiver not only as a conduit for patient care but as a patient in their own right, deserving of attention, empathy and resources [2]. This study is limited by its cross-sectional design, which restricts the ability to draw causal inferences. Furthermore, reliance on self-report tools may introduce subjective bias and the sample may not represent the full diversity of socioeconomic or cultural contexts. Future research should explore longitudinal outcomes and test targeted interventions for high-risk caregiver groups. The study provides compelling evidence that higher levels of psychological distress, including anxiety, depression and stress, are significantly associated with lower quality of life across physical, psychological, social and environmental domains among caregivers of children with cerebral palsy.

## Conclusion:

The strong interconnection between psychological distress and impaired quality of life in caregivers of children with CP is shown. The severity of the child's motor impairment further exacerbates these challenges. These results emphasize the pressing need for supportive policies and caregiver-focused interventions to promote physical, psychological and social well-being among this vulnerable population. Prioritizing caregiver health is essential not only for their benefit but also for optimizing the care and developmental outcomes of children with cerebral palsy.
